# An exploratory study to assess the influence of schistosomiasis on the occurrence of dengue virus in Madagascar

**DOI:** 10.1186/s40249-025-01370-0

**Published:** 2025-09-26

**Authors:** Jana Christina Hey, Tahinamandranto Rasamoelina, Anjarasoa Ravo Razafindrakoto, Nantenaina Matthieu Razafindralava, Zaraniaina Tahiry Rasolojaona, Stephanie Leyk, Sreejith Rajasekharan, Lucas Wilken, Tiana Randrianarisoa, Tojo Rémi Rafaralahivoavy, Jacques Hainasoa, Raphael Rakotozandrindrainy, Njary Randriamampionona, Norbert Georg Schwarz, Anna Jaeger, Aaron Remkes, Jean-Marc Kutz, Pia Rausche, Irina Kislaya, Valentina Marchese, Christa Ehmen, Christina Deschermeier, Jürgen May, Lidia Bosurgi, Rivo Andry Rakotoarivelo, Pietro Scaturro, Daniela Fusco

**Affiliations:** 1 Research Group: Implementation Research, Bernhard Nocht Institute for Tropical Medicine, Hamburg, Germany; 2 Research Unit: Systems Arbovirology, Leibniz Institute of Virology, Hamburg, Germany; 3 German Center for Infection Research, Hamburg-Borstel-Lübeck-Riems, Hamburg, Germany; 4 Centre d’ Infectiologie Charles Mérieux, University of Antananarivo, Antananarivo, Madagascar; 5 I. Department of Medicine, University Medical Center Hamburg-Eppendorf and Hamburg Center for Translational Immunology, Hamburg, Germany; 6 Department of Protozoa Immunology, Bernhard Nocht Institute for Tropical Medicine, Hamburg, Germany; 7 Department of Infectious Diseases, Infectious Diseases Service, University Hospital Tambohobe, Fianarantsoa, Madagascar; 8 Radiology Department, University Hopsital of Andrainjato, Fianarantsoa, Madagascar; 9 Department of Infectious Diseases, University of Fianarantsoa Andrainjato, Fianarantsoa, Madagascar; 10 Department of Microbiology and Parasitology, University of Antananarivo, Antananarivo, Madagascar; 11 Department of Infectious Disease Epidemiology, Bernhard Nocht Institute for Tropical Medicine, Hamburg, Germany; 12 Diagnostics Development Laboratory, Bernhard Nocht Institute for Tropical Medicine, Hamburg, Germany; 13 Panadea Diagnostics GmbH, Hamburg, Germany; 14 Tropical Medicine I, University Medical Center Hamburg-Eppendorf, Hamburg, Germany

**Keywords:** Dengue virus, Schistosomiasis, Seroprevalence, Madagascar, Co-infection, Parasitic infections, Arboviruses

## Abstract

**Background:**

Dengue virus (DENV) is the most prevalent mosquito-borne virus worldwide, with approximately half of the world’s population at risk of infection. Although it has been shown that parasitic infections can influence viral co-infections the role of schistosomiasis has not yet been explored. The objective of this exploratory study was to investigate the influence of schistosome infection on DENV infection in Madagascar.

**Methods:**

Between March 2020 and October 2022 we recruited participants in the regions of Boeny and Atsinanana to assess the seroprevalence of DENV in the country using highly specific tests for the detection of IgG antibodies and investigated the influence of schistosome infections on DENV infections through a plaque reduction neutralisation test (PRNT). For this, additional participants were recruited in Haute Matsiatra between July 2022 and March 2023, Poisson regression models were used to assess the association of the PRNT results with schistosome infections.

**Results:**

For the first time, we report a low seroprevalence of DENV (up to 3.3%) in areas with a high prevalence (> 50%) of schistosome infection. Additionally, we could demonstrate that sera derived from schistosome-infected individuals exert a significant antiviral activity against DENV infection (up to 27.5%). A Poisson regression analysis revealed that, among the possible factors assessed, the schistosome infection status was the only factor associated with the inhibitory effects against DENV infection in the PRNT. Finally, we could observe that highest IgE level were found in participants showing the greatest reduction in viral infection in the PRNT.

**Conclusions:**

Our data suggest that schistosomiasis might play a protective role against DENV infections. These findings offer new perspectives regarding how chronic parasitic infections affect the dynamics of DENV infections in Africa.

**Graphical Abstract:**

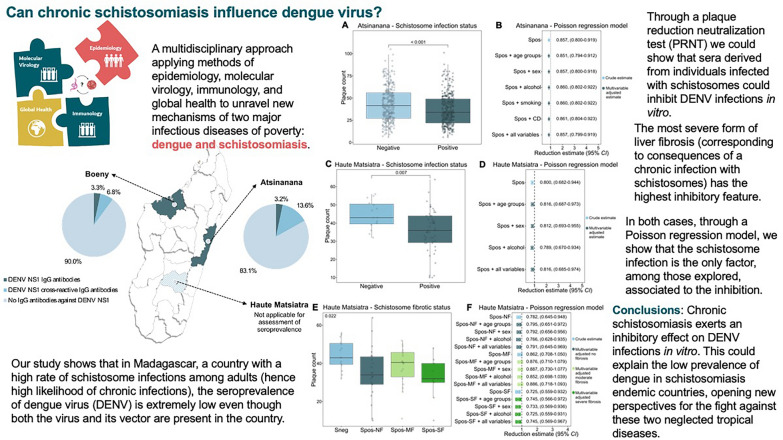

**Supplementary Information:**

The online version contains supplementary material available at 10.1186/s40249-025-01370-0.

## Background

Dengue virus (DENV) is the most prevalent mosquito-borne virus worldwide and has historically not been considered endemic in Africa, despite the presence of the main arthropod vectors on the continent [1]. Approximately half of the world’s population is at risk of infection, and more than 400 million infections are estimated to occur each year, accounting for approximately 1.1 million disability-adjusted life-years (DALYs) [2–5]. Overall, the true burden of dengue and other arboviral diseases in Africa is still unclear.

Parasitic infections modulate immune responses, and have been described to influence the outcome of secondary infections, including viral infections [6–9]. Among the hypothesised factors influencing the host immune system, the T helper cell 2 (Th2) response elicited by parasitic infections [including the increased production of interleukin-4 (IL-4), IL-13, and immunoglobulin E (IgE)] has been proposed to play an important role [9]. Interestingly, a review on the seroprevalence of DENV infections in Africa reported high heterogeneity across the continent and identified a trend of the highest prevalence in those countries where schistosomiasis levels are the lowest [10, 11].

Schistosomiasis is caused by helminths of the genus *Schistosoma* [11, 12]. Approximately 779 million people are at risk of infection worldwide, and over 250 million people are infected, with the majority living in sub-Saharan Africa [11]. This results in an estimated global disease burden of about 1.7 million DALYs [11, 13], with the highest burden of the disease associated mostly with the chronic course of infection [11, 14]. While the initial response to the parasite is mainly a type 1-mediated response [characterized by IFN-γ, tumor necrosis factor (TNF), and IL-12 secretion], high modulation of dendritic cells and macrophage activation contribute to a switch toward a type 2-response/regulatory phase [15–17]. Monocytes, dendritic cells, and macrophages are among the primary target cells of DENV [18, 19].

Madagascar is one of the countries with the highest prevalence of schistosomiasis globally, with over 50% of the population infected [11] and a high likelihood of chronic infections. Mosquito-borne diseases, such as malaria and dengue, have been described only in coastal areas. However, reports of DENV infections in Madagascar are scarce, despite conducive climate conditions and the presence of multiple competent arthropod species. To date, two major DENV outbreaks have been described in 2006 and 2020 [20, 21].

The aim of this exploratory study was to investigate the potential influence of schistosome infections in endemic areas on the occurrence of DENV infections. The specific objectives were (i) to assess the seroprevalence of DENV in Madagascar and (ii) to evaluate the influence of schistosome infections on DENV infections.

## Methods

### Study setting and data collection

Participants were recruited from the regions Boeny (16°06′50″S 46°45′24″E), Atsinanana (19°19′49"S 48°58′49"E), and Haute Matsiatra (21°26′38″S 47°05′16″E). Eligibility criteria for all studies were informed consent, willingness to comply with the protocol, and age ≥ 18 years for Boeny and Haute Matsiatra, or ≥ 5 years for Atsinanana. A general exclusion criterion was the history of epilepsy or convulsive episodes. For Boeny, the additional exclusion criteria of fever (temporary exclusion), a history of transfusion or congenital anaemia were applied; for Atsinanana and Haute Matsiatra, a history of bleeding/haemorrhage, current pregnancy as well as current suspected and/or confirmed coronavirus disease 2019 (COVID-19) infection and COVID-19 vaccination within the past two weeks before recruitment. For the regions Boeny [22] and Atsinanana [23], recruitment was previously described in detail. Participants were recruited from a primary healthcare centre in Ankazomborona in the Boeny region (March 2020–January 2021) and through home-based surveys in Vatomandry in the Atsinanana region (July–October 2022). From each participant 9 ml of venous blood was collected, and serum aliquots were stored at −80 ℃. In Haute Matsiatra (July 2022–March 2023), participants with different degrees of hepatosplenic schistosomiasis [schistosome-negative (Sneg), schistosome-positive (Spos) without liver fibrosis (Spos-NF), Spos with moderate liver fibrosis (Spos-MF), and Spos with severe liver fibrosis (Spos-SF)] were recruited at the main hospital [*Centre Hospitalier Universitaire*(*CHU*) *Tambohobe Fianarantsoa*], with pre-recruitment conducted at the primary level of healthcare and subsequent referral to CHU. A urine sample was taken and a rapid point-of-care circulating cathodic antigen test (POC-CCA) was performed to screen for schistosome infections. Disease severity in infected participants was assessed by staging the degree of liver fibrosis via ultrasound and segregating participants into the four corresponding groups. From each participant 5 ml of venous blood was collected to obtain an aliquot of serum, and 9 ml of venous blood was collected to obtain an aliquot of plasma, which were stored at −80 °C. All the samples were shipped on dry ice to the Bernhard Nocht Institute for Tropical Medicine (BNITM), Hamburg, Germany, where they were stored at −80 °C until further use. Background characteristics such as socio-demographic information, clinical history, and personal habits were provided in a pseudonymised format and later merged with the test results.

At all the study sites, data were collected through case report forms (CRFs), which were administered via face-to-face interviews, either directly through eCRFs or by double data entry, according to data quality standards. The CRF data were managed using REDCap^®^ electronic data capture tools version 15.0.33 (Vanderbilt University, Nashville, USA) hosted at the BNITM. The questionnaire was developed ad-hoc for the studies in collaboration with all the implementing partners. After finalisation, a pilot test was run during the training sessions, and additional adaptations were included if needed.

### Sample size considerations

Sample size calculations were based on the premise that this study represents a secondary analysis of data originally collected for other objectives.

For the seroprevalence analysis, on the basis of previously available data from Madagascar, we assumed a DENV seroprevalence of 10%. To detect a 5% difference in the prevalence of DENV between subgroups with 80% power, we estimated the minimum required sample size to be 864 participants. Given the availability of overall 1478 participants, we included this total number.

To analyse the average reduction in plaque counts, the minimum required sample size was estimated using the non-parametric Guenther method for the Mann–Whitney test [24], as we anticipated a skewed distribution of plaque counts. To achieve 80% power for detecting small (Cohen's d = 0.2), medium (d = 0.5), and large (d = 0.8) effect sizes, a minimum of 412, 67, and 27 participants per group, respectively, were required. Sample size calculations were conducted using the "pwrss" package in R software version 4.4.2 (R Core Team, Vienna, Austria).

### Laboratory testing

Different methodologies have been applied to detect the presence of schistosomes and previous DENV infections (diagnostic assays), whereas experimental in vitro assays have been established to assess the influence of schistosome infections on DENV infections (experimental assays).

#### Diagnostic assays

*Schistosome PCR testing.* DNA extraction and PCR were performed as previously described [22]. Briefly, DNA was extracted from 1 ml serum using the QIAamp MinElute ccfDNA Mini Kit, following the manufacturer’s instructions (Qiagen, Hilden, Germany). The extracted DNA was stored at −20 °C until further use. Semi-quantitative PCR (qPCR), which is based on a previously published protocol [25], was performed to simultaneously detect *S. mansoni* and *S. haematobium* infections. The readout resulted from the RotorGene 6000 Software v.7.87 (Qiagen, Hilden, Germany). Results with a clean sigmoid curve were considered positive.

*POC-CCA testing.* POC-CCA was performed on site according to the manufacturer’s instructions (Rapid Medical Diagnostics, Pretoria, South Africa) to detect schistosome infections. Two drops (100 µl) of urine were transferred to the POC-CCA test cassette and the results were read after 20 min.

*Pan-DENV IgG ELISA*. Sera were screened with the pan-DENV (NS1) IgG ELISA (Panadea Diagnostics, Hamburg, Germany) to identify samples with antibodies targeting DENV NS1. Afterwards, samples that tested positive in the screening were subjected to the pan-DENV (NS1) IgG *SE* ELISA (Panadea Diagnostics, Hamburg, Germany). The specificity enhancer in this kit suppresses signals from cross-reactive orthoflavivirus antibodies against DENV NS1, leaving only dengue-type-specific signals. Sera were diluted 1∶51 and incubated overnight at 4 °C in a sealed, moist environment with horseradish peroxidase (HRP)-labelled recombinant DENV NS1 antigen (serotypes 1–4), following the manufacturer’s instructions. The optical density (OD) was assessed at 450/620 nm, and the difference in the OD_450_–OD_620_ was calculated for each sample. The positivity threshold for samples from Boeny was manually defined as OD > 0.14 and as OD > 0.175 for samples from Atsinanana. All samples that tested positive with the pan-DENV (NS1) IgG ELISA were subjected to the pan-DENV (NS1) IgG *SE* ELISA. Samples that tested positive according to the pan-DENV (NS1) IgG *SE* ELISA were ultimately considered positive for dengue-type-specific antibodies. Samples that tested positive according to the pan-DENV (NS1) IgG ELISA but negative according to the pan-DENV (NS1) IgG *SE* ELISA were considered as being positive for DENV NS1 cross-reactive antibodies.

#### Experimental assays

*Plaque reduction neutralization test (PRNT).* Non-heat-inactivated serum was diluted 1∶8 with Dulbecco's Modified Eagle Medium, mixed with an equal volume of DENV2 (strain UVE/DENV-2/2018/RE/47099) and incubated for 1 h at 37 °C. The virus concentration used was adjusted to a range of 10–100 plaque forming units (PFU)/well, with 10 being the minimum plaque count when the DENV complex-reactive mouse monoclonal antibody D1-11(3) (IgG2a; GeneTex, Irvine, USA) was used as a positive neutralization control (final concentration: 1 µg/ml). The virus-serum mixture was transferred onto a Vero E6 monolayer (2.5 × 10^5^ cells/24-well) to allow infection for 1 h at 37 °C. The mixture was aspirated, and the cells were overlaid with 1.5% carboxymethyl cellulose in Minimum Essential Medium supplemented with 2% fetal bovine serum and incubated for six days at 37 °C in 5% CO_2_. Afterwards, the cells were fixed with 10% formaldehyde and stained with 1% crystal violet solution as previously described [26]. The plaque count for each sample was assessed and used for further analysis.

*Cytokine profiling.* Sera were subjected, in duplicate, to fluorescent bead measurement of cytokines and chemokines using the Human T Helper Cytokine Panel V02 (Cat # 741028), the Human Anti-Virus Response Panel (Cat # 740390), and the Human Anti-Virus Response Panel V02 (Cat # 741270), following the manufacturer’s instructions (BioLegend, San Diego, USA). The standard curves were optimized automatically by the software LEGENDplex Data Analysis Software Suite version 2025-05-01 (Qognit, San Carlos, USA) and verified manually.

*IgE ELISA.* The plasma samples were subjected to the Human IgE ELISA (Invitrogen, Waltham, USA), following the manufacturer’s instructions. Sample duplicates were bound to antibodies absorbed onto the microwells, before the addition of HRP-conjugated anti-human IgE antibodies. A colourful signal was measured at OD_450_. Sample IgE concentrations were determined via a standard curve for reference.

### Statistical analysis

For numerical variables, medians and interquartile ranges (IQRs) are reported. For categorical variables, absolute and relative frequencies are reported. Seroprevalence rates with 95% confidence intervals (*CIs*) and crude prevalence odds ratios (*cPORs*) with 95% *CIs* were estimated using the R package epiR. Chi-square or Fisher’s exact tests were used to compare estimates between subgroups. The *CIs* of the median of cytokine concentrations were computed using the R package DescTools.

Comparisons of plaque counts, cytokine and IgE concentrations between groups were made via non-parametric Mann-Whitney-Wilcoxon or Kruskal-Wallis tests when applicable. Non-parametric tests were applied to provide more robust results accounting for small sample sizes and skewed distributions of outcome variables. Boxplots were used for graphical representation of plaque counts and IgE concentrations.

A multivariable Poisson regression model with correction for overdispersion was used to assess the effect of schistosome positivity on plaque counts and the influence of other covariables on this estimate. The percentage of reduction in the plaque count was calculated as (1–RR) × 100%. All analyses were performed using R software version 4.4.2 (R Core Team, Vienna, Austria).

## Results

### Description of the study population

**Fig. 1:**
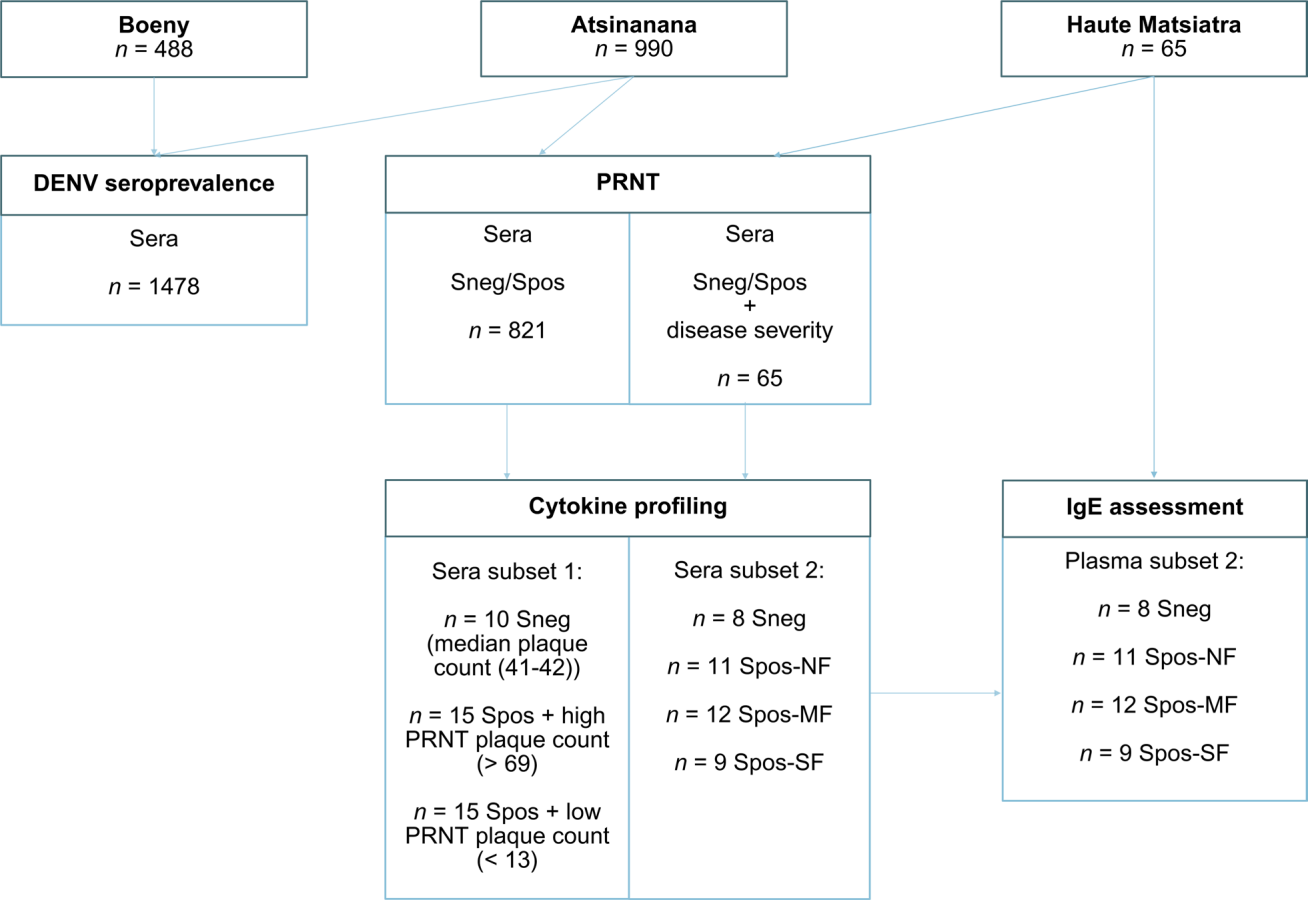
Inclusion flowchart of participants and samples for the performed experiments. *DENV* dengue virus, *Sneg* schistosome-negative, *Spos* schistosome-positive, *PRNT* plaque reduction neutralisation test, *Spos-NF* schistosome-positive without liver fibrosis, *Spos-MF* schistosome-positive with moderate liver fibrosis, *Spos-SF* schistosome-positive with severe liver fibrosis

Overall, this study included 1543 serum and 65 plasma samples from participants in the three regions of Madagascar (Fig. 1).

The study sample characteristics for the three regions involved are reported in Table 1. The population of Boeny (*n* = 488) was characterised by 55% females (*n* = 266), a median age of 28 years (IQR: 21–40) and mostly farmers (77%, *n* = 372). The prevalence of schistosome infection was 65% (*n* = 315), as assessed by qPCR. The population of Atsinanana (*n* = 990) was characterised by 56% females (*n* = 557), a median age of 42 years (IQR: 32–54) and mostly farmers (59%, *n* = 563). Chronic diseases were self-reported by 5% (*n* = 51) of the participants. The prevalence of schistosome infection was 55% (*n* = 548), assessed by qPCR. Alcohol consumption and smoking were reported by 36% (*n* = 358) and 9% (*n* = 87) of the participants, respectively. The population of Haute Matsiatra (*n* = 65) was characterised by 68% females (*n* = 44) and a median age of 38 years (IQR: 27–49). Farming was reported by only 20% (*n* = 13) of the participants. Alcohol consumption was reported by 11% (*n* = 7). The prevalence of schistosome infection was 77% (*n* = 50) on the basis of the POC-CCA results. The participants were also assessed for other infections (HBV, HCV, or HIV), but only one participant was co-infected with HBV.

**Table 1:** Background characteristics of the study sample, segregated by recruitment region

	Boeny (*n* = 488)	Atsinanana (*n* = 990)	Haute Matsiatra (*n* = 65)
Age, years			
Median (IQR)	28 (21–40)	42 (32–54)	38 (27–49)
Age groups, years			
0–17	NA	23 (2%)	NA
18–29	255 (52%)	174 (18%)	19 (29%)
30–44	137 (28%)	346 (35%)	21 (32%)
45–59	61 (13%)	289 (29%)	19 (29%)
60 +	35 (7%)	158 (16%)	6 (9%)
Sex		*n* = 988^*^	
Female	266 (55%)	557 (56%)	44 (68%)
Male	222 (45%)	431 (44%)	21 (32%)
Occupation	*n* = 484^*^	*n* = 961^*^	
Non-farmer	112 (23%)	398 (41%)	52 (80%)
Farmer	372 (77%)	563 (59%)	13 (20%)
Alcohol consumption			
No	NA	632 (64%)	58 (89%)
Yes	NA	358 (36%)	7 (11%)
Smoking			
No	NA	903 (91%)	NA
Yes	NA	87 (9%)	NA
Chronic disease (self-reported)^†^		*n* = 987^*^	
No	NA	936 (95%)	NA
Yes	NA	51 (5%)	NA
Schistosome infection status^§^			
Negative	173 (35%)	442 (45%)	15 (23%)
Positive	315 (65%)	548 (55%)	50 (77%)
Degree of liver fibrosis			
Sneg	NA	NA	15 (23%)
Spos-NF	NA	NA	22 (34%)
Spos-MF	NA	NA	18 (28%)
Spos-SF	NA	NA	9 (14%)
No assessment	NA	NA	1 (1%)
Test performed			
Seroprevalence	488 (100%)	990 (100%)	NA
PRNT	NA	821 (83%)^¶^	65 (100%)

*IQR* interquartile range, *Sneg* schistosome-negative, *Spos* schistosome-positive, *Spos-NF* schistosome-positive without liver fibrosis, *Spos-MF* schistosome fibrosis with moderate fibrosis, *Spos-SF* schistosome-positive with severe fibrosis, *PRNT* plaque reduction neutralisation test, *qPCR* semi-quantitative polymerase chain reaction, *POC-CCA* point-of-care circulating cathodic antigen

^*^Participants with missing information were not included

^†^Self-reported chronic diseases included asthma, allergy, cough, dermatosis, diabetes, elephantiasis, high blood pressure, joint pain, kidney disease, memory loss, palpitations, rheumatism, stomach ache, tuberculosis, and vaginal discharge

^§^The schistosome infection status was assessed using qPCR for samples collected in Boeny and Atsinanana. POC-CCA was used to assess samples collected in Haute Matsiatra

^¶^Due to limited availability of samples, PRNT was only performed on a smaller subset

### Assessment of DENV IgG seroprevalence in the regions of Boeny and Atsinanana

**Fig. 2:**
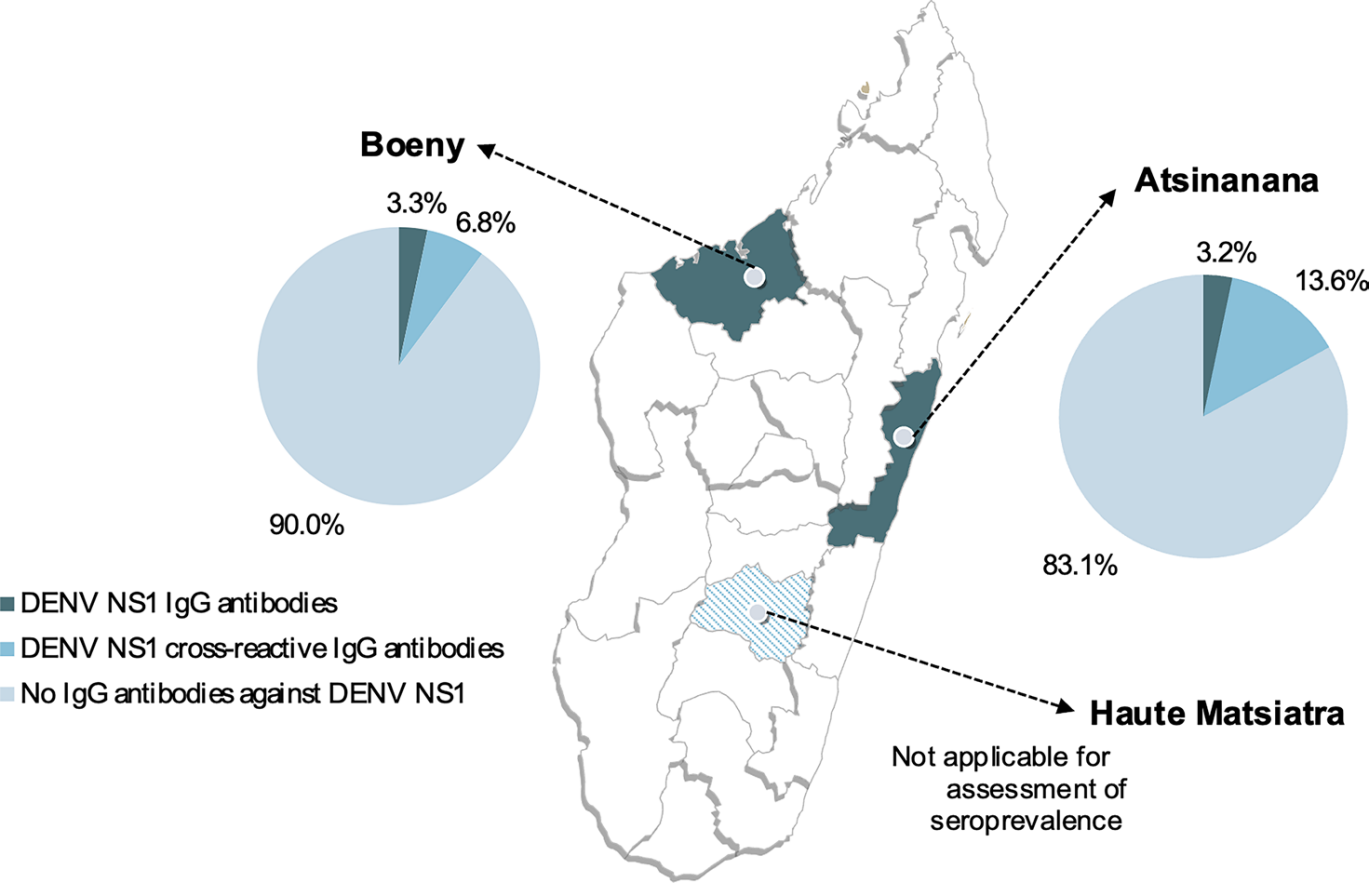
Study sites in Madagascar for DENV seroprevalence and PRNT. The prevalence of DENV NS1 IgG antibodies was assessed in two coastal regions of Madagascar (Boeny, *n* = 488; Atsinanana, *n* = 990); seroprevalence was not assessed for the third region (Haute Matsiatra, *n* = 65), as no relevant DENV transmission has been reported in the mountain areas of Madagascar. *DENV* dengue virus, *PRNT* plaque reduction neutralisation test

Using the pan-DENV (NS1) IgG ELISA, a prevalence of DENV NS1-reactive IgG antibodies of 10.0% (95% *CI:* 7.5–13.1) and 16.9% (95% *CI*: 14.6–19.4) was detected in Boeny and Atsinanana, respectively. When the results were adjusted with the pan-DENV (NS1) IgG *SE* ELISA, a DENV-specific seroprevalence of 3.3% (95% *CI:* 1.9–5.3) and 3.2% (95% *CI:* 2.2–4.5) was detected in both regions, respectively (Fig. 2). Therefore, cross-reactive antibodies against DENV NS1 were detected in 6.8% (*n* = 33) and 13.6% (*n* = 135) of the participants, respectively.

The DENV seroprevalence estimates stratified by various variables with *cPOR* are reported in Table 2. The DENV seroprevalence did not significantly differ by sex, age group, or region of origin, based on chi-square and Fisher’s exact tests. However, the seroprevalence of DENV was significantly lower among farmers with a *cPOR* of 0.5 (95% *CI:* 0.3–0.9). The *cPOR* of DENV seroprevalence among schistosome-infected participants was 0.9 (95% *CI:* 0.5–1.6), which was lower than that among non-infected participants.

**Table 2:** DENV seroprevalence and crude prevalence odds ratios

	DENV positive, *n*	Total, *n*	Prevalence (%)	*cPOR* (95% *CI*)	*P*-value
Age groups^*^					
0–17	2	23	8.7 (1.1–28.0)	3.4 (0.7–16.3)	0.145
18–29	15	429	3.5 (2.0–5.7)	1.3 (0.6–2.8)	0.565
30–44	13	483	2.7 (1.4–4.6)	Ref	
45–59	14	350	4.0 (2.2–6.6)	1.5 (0.7–3.3)	0.325
60 +	4	193	2.1 (0.6–5.2)	0.8 (0.3–2.4)	0.790
Sex					
Female	26	823	3.2 (2.1–4.6)	Ref	
Male	22	653	3.4 (2.1–5.1)	1.1 (0.6–1.9)	0.821
Occupation					
Non-farmer	24	510	4.7 (3.0–6.9)	Ref	
Farmer	22	935	2.4 (1.5–3.5)	0.5 (0.3–0.9)	**0.015**
Schistosome infection status					
Negative	21	615	3.4 (2.1–5.2)	Ref	
Positive	27	863	3.1 (2.1–4.5)	0.9 (0.5–1.6)	0.760
Region					
Boeny	16	488	3.3 (1.9–5.3)	Ref	
Atsinanana	32	990	3.2 (2.2–4.5)	1.0 (0.5–1.8)	0.962

*DENV* dengue virus, *cPOR* crude prevalence odds ratio, *CI* confidence interval, *Ref* reference

^*^P-value: Fisher’s exact test

The seroprevalence in Haute Matsiatra was not assessed as dengue, and other mosquito-transmitted infections, are neither regularly reported nor expected to occur in this area because of the altitude of the region.

### Effects of soluble serum factors from schistosome-infected participants on DENV infection

**Fig. 3:**
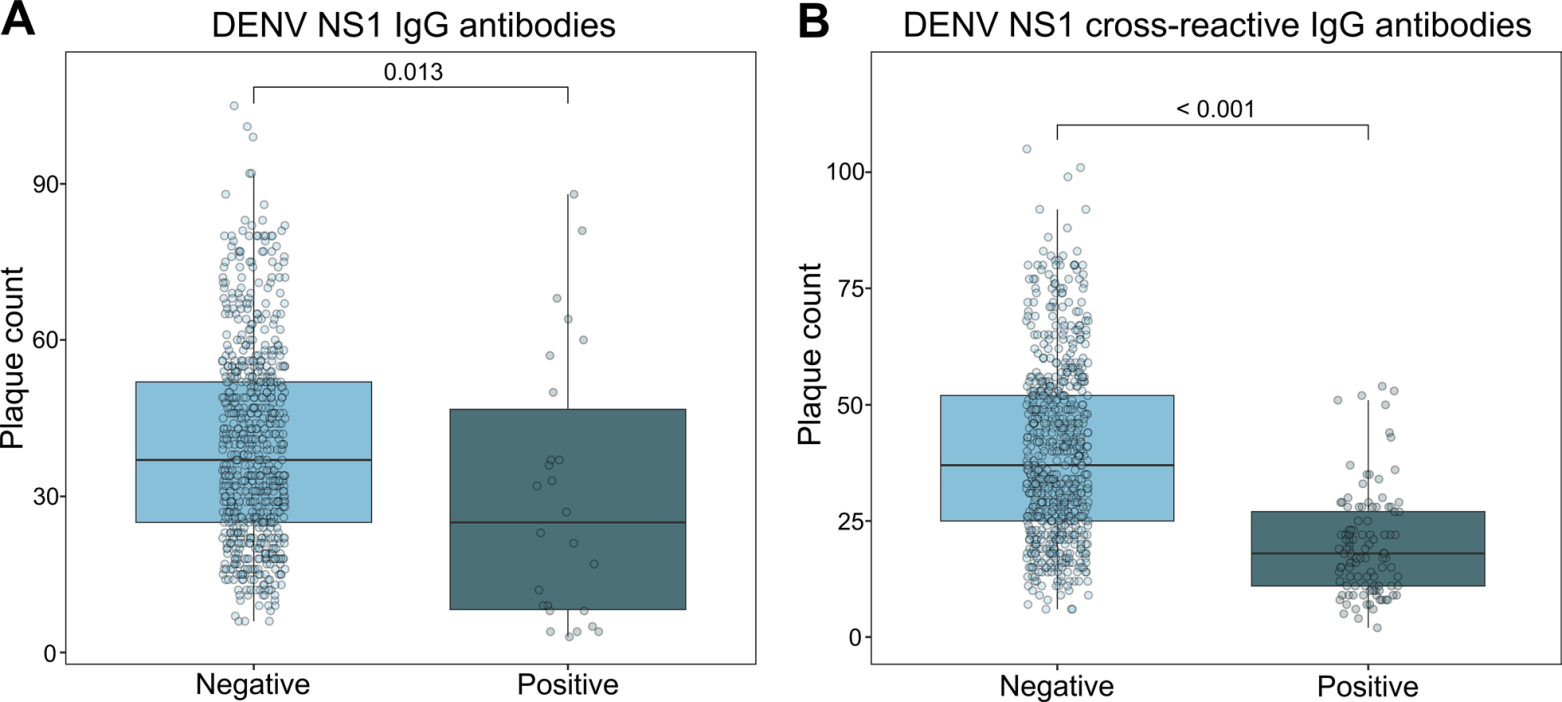
Verification of PRNT establishment. PRNT results for 713 sera (exclusion of *n* = 108 with DENV NS1 cross-reactive IgG antibodies), depicted by plaque count of samples and stratified by presence of DENV NS1 IgG antibodies (*n* positive = 26, *n* negative = 687, *P* = 0.013, estimated with Mann-Whitney-Wilcoxon test; median positive = 25, median negative = 37) (panel A). PRNT results for 795 sera (exclusion of *n* = 26 with DENV-specific NS1 IgG antibodies), depicted by plaque count and stratified by presence of DENV NS1 cross-reactive IgG antibodies (*n* positive = 108, *n* negative = 687, *P* < 0.001, estimated with Mann-Whitney-Wilcoxon test; median positive = 18, median negative = 37) (panel B). *PRNT* plaque reduction neutralisation test, *DENV* dengue virus

A PRNT was performed on a total of 886 participants divided into two groups. The first group included 821 participants from Atsinanana, on which the seroprevalence was assessed. As expected, sera from individuals with pre-existing DENV antibodies (*n* = 26) or cross-reactive antibodies to DENV NS1 (*n* = 108) presented a significant reduction in the PRNT (Fig. 3). To rule out confounding effects arising from pre-existing immunity, these sera were removed from further analysis.

**Fig. 4:**
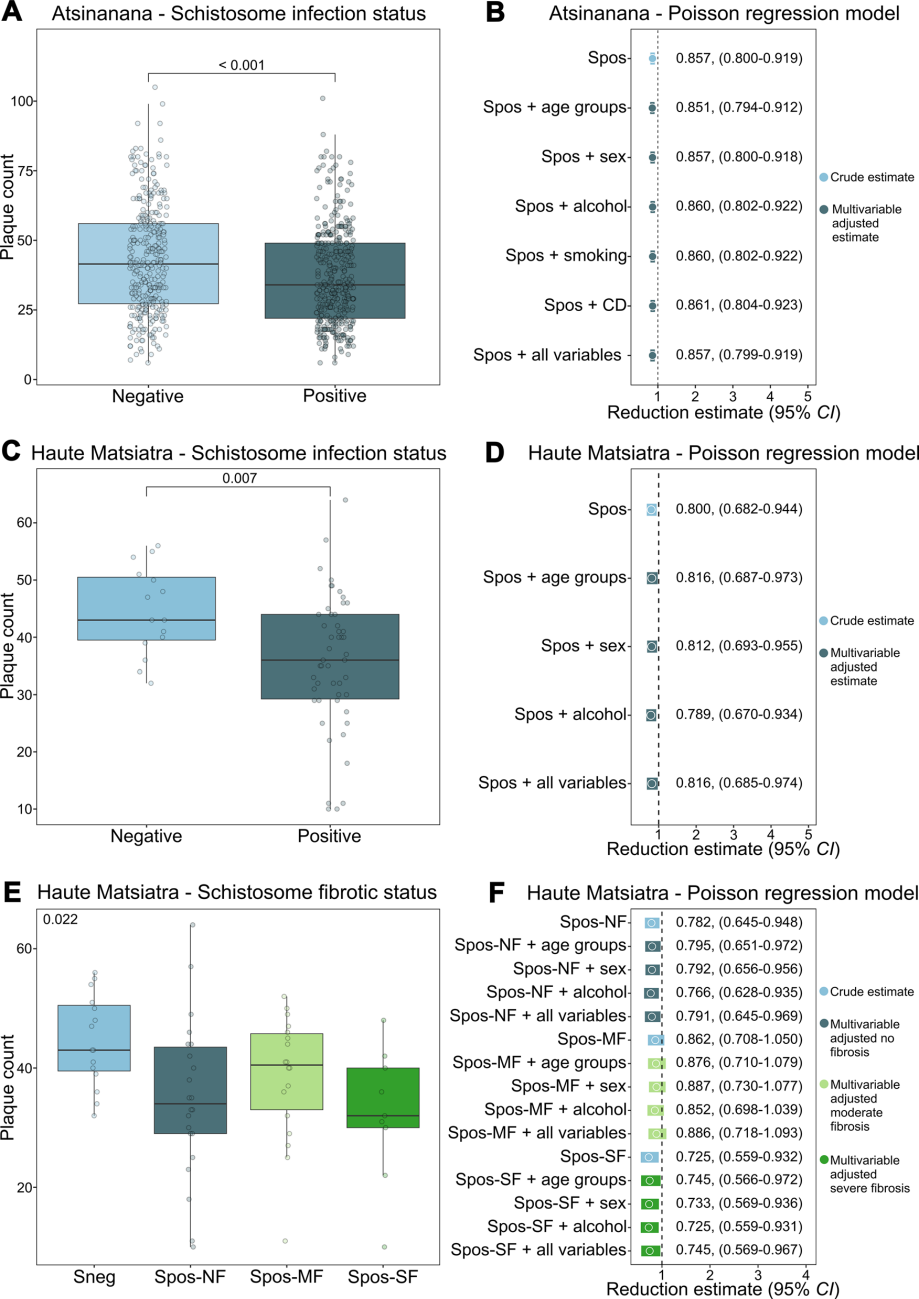
The effect of Spos sera from Atsinanana and Haute Matsiatra on DENV infections. PRNT results for 687 sera, depicted by plaque count and stratified by presence of schistosome infection (*n* positive = 365, *n* negative = 322, *P* < 0.001, estimated with Mann-Whitney-Wilcoxon test; median positive = 34, median negative = 41.5) (panel **A**). PRNT results for 65 sera, depicted by plaque count and stratified by presence of schistosome infection (*n* positive = 50, *n* negative = 15, *P* = 0.007, estimated with Mann-Whitney-Wilcoxon test; median positive = 36, median negative = 43) (panel **C**). PRNT results for 65 sera, depicted by plaque count and stratified by presence of schistosome infection and degree of liver fibrosis (*n* Sneg = 15, *n* Spos-NF = 22, *n* Spos-MF = 18, *n* Spos-SF = 9, *P* = 0.022, estimated with Kruskal-Wallis test; median Sneg = 43, median Spos-NF = 33, median Spos-MF = 40.5, median Spos-SF = 32) (panel **E**). Multivariable Poisson regression models with correction for overdispersion for the *Schistosoma* positivity estimate (panel **B**, panel **D**) and *Schistosoma* positivity with degrees of liver fibrosis (panel **F**) (crude and adjusted for relevant covariables) depicted with 95% *CI*; Sneg = reference category. *Spos* schistosome-positive, *DENV* dengue virus, *PRNT* plaque reduction neutralisation test, *Spos-NF* schistosome-positive without liver fibrosis, *Spos-MF* schistosome-positive with moderate fibrosis, *Spos-SF* schistosome-positive with severe fibrosis, *CI* confidence interval, *CD* chronic disease

We then observed a statistically significant plaque count reduction of 14.3% (RR: 0.857; 95% *CI*: 0.800–0.919) among schistosome-infected participants compared with Sneg (Fig. 4a). A Poisson regression analysis revealed that, among the possible factors assessed, the schistosome infection status was the only factor associated with the inhibitory effects against DENV infection in the PRNT (Fig. 4b).

### Effects of soluble serum factors from participants with different degrees of liver fibrosis on DENV infection

In the second group, which included 65 participants from Haute Matsiatra, we assessed whether sera from participants, affected by varying degrees of fibrosis, would have a differential influence on the assay. A plaque count reduction of 20.0% (RR: 0.800; 95% *CI*: 0.682–0.944) was observed among infected participants in the PRNT (Fig. 4c). In this group, the Poisson regression analysis confirmed that schistosome infection was the only factor significantly associated with the plaque count reduction (Fig. 4d). When the samples were stratified by the degree of liver fibrosis, a non-linear association with disease severity was observed (*P* = 0.022, Fig. 4e). The greatest antiviral effects in the PRNT were observed between Sneg and Spos-NF at 21.8% (RR: 0.782; 95% *CI:* 0.645–0.948), and Sneg and Spos-SF at 27.5% (RR: 0.725; 95% *CI:* 0.559–0.932). Collectively, these data indicated that the degree of liver fibrosis was the only factor significantly associated with a reduction in DENV infectivity in vitro (Fig. 4f).

### Contribution of secreted cytokines and IgE to the antiviral activity of schistosome-positive sera

Next, we aimed to characterise the soluble factors potentially associated with the observed protective effects of schistosomiasis sera against DENV infection. Given the quantity and type of samples needed, we quantitatively assessed the levels of a selected panel of cytokines related to the chronic immune response to schistosomes (e.g., IL-4, IL-13, and IL-5) and DENV infections (e.g., IFN-α2 and TNF-α). Furthermore, we targeted IgE due to its specific role in parasitic infections.

First, a subset (*n* = 40) of sera from Atsinanana with comparable socio-demographic characteristics was selected on the basis of the PRNT results: (i) Sneg samples with plaque counts similar to the median (*n* = 10), (ii) Spos samples with the highest plaque counts (*n* = 15), and (iii) Spos samples with the lowest plaque counts (*n* = 15). Few differences among the cytokine profiles of the three groups were observed (Supplementary Table 1). Upon combining the two Spos groups, we observed a mild decrease in the levels of pro-inflammatory cytokines (IL-1β, IFN-α2, IL-6, and TNF-α) among the Spos sera. However, statistically significant differences were identified only for IFN-α2 (*P* = 0.014) and TNF-α (*P* = 0.029) (Supplementary Table 2). Second, a subset of sera from Haute Matsiatra (*n* = 40) was selected for further testing: (i) Sneg samples (*n* = 8), (ii) Spos-NF samples (*n* = 11), (iii) Spos-MF samples (*n* = 12), and (iv) Spos-SF samples (*n* = 9). No significant differences were observed in the cytokine concentrations when the four groups were analysed separately or when the three Spos groups were combined, based on Mann-Whitney-Wilcoxon and Kruskal-Wallis tests. However, a decrease in TNF-α with increasing disease severity was observed (Supplementary Table 3).

IgE levels were assessed in the same subset of plasma from Haute Matsiatra (*n* = 40). Overall, the median IgE level steadily increased with increasing disease severity in schistosomiasis participants (Supplementary Fig. 1). The highest median concentration was found in the Spos-SF group. In the same group, the greatest reduction in viral infection was observed in the PRNT.

## Discussion

This study reports for the first time a low seroprevalence of DENV (up to 3.3%) in areas with a high prevalence of schistosome infection (> 50%) in multiple regions of Madagascar and a significant antiviral activity against DENV infection in vitro in sera derived from schistosome-infected individuals (up to 27.5%).

Compared with previous studies in Madagascar, the lower seroprevalence of DENV detected in this study [27, 28] can be explained by the different methodologies used. The seroprevalence assessment in our study is based on a recently marketed Fc-receptor-based assay, allowing for improved pan-DENV serology. This assay enables differential detection of IgG antibodies elicited by contact with different orthoflaviviruses. Using this approach, we demonstrate that 6–14% of all DENV seropositive tests are due to cross-reactive antibodies against DENV NS1. This finding indicates the presence of other orthoflaviviruses circulating in the area. Hence, we speculate that the differences observed in our study might be due to the broad cross-reactivity of widely used tests for the detection of IgG antibodies against multiple orthoflaviviruses. The specific assays used here, in contrast, provide a more accurate and realistic assessment of the DENV seroprevalence in the area. We observed a tendency toward a lower DENV prevalence in schistosome-infected individuals. Additionally, we observed a decreased prevalence of DENV in farmers. As a population in direct and continuous contact with stagnating waters, farmers are expected to be at high risk of DENV infection [29], which contrasts our findings. Nevertheless, farmers were described in previous studies to be among the groups with the highest prevalence of schistosomiasis [23, 30].

Importantly, our results strongly support a protective effect of sera from schistosome-infected individuals (reduction of 14.3%), and those presenting with no fibrosis or high disease severity (reduction of up to 27.5%), against DENV infections in vitro. Although the PRNT is conventionally considered the gold standard of serological tests for characterising and quantifying circulating levels of DENV-specific neutralising antibodies [31, 32], its experimental design allows the visualisation of the influence of other soluble factors on viral infectivity [33]. This feature supports its validity for use in our study. Collectively, these data suggest that the degree of liver fibrosis, specifically either no fibrosis or severe fibrosis, which represent the two extremes of the immune response spectrum during *Schistosoma* infections, is more likely associated with the secretion of soluble mediators that confer protection against DENV infection in vitro. In contrast, moderate fibrosis does not show this association.

Analysis of IgE levels across different participant groups revealed increased concentrations in the Spos-SF group, which also presented the greatest antiviral activity in the PRNT group, suggesting a possible connection between increased IgE levels and anti-DENV activity.

To our knowledge, this is one of the first studies leveraging a well-controlled and stratified epidemiological survey in an endemic country, with a targeted phenotypic approach to characterise the effect of pre-existing schistosome infections on the disease outcome of acute viral infections. Importantly, leveraging a large cohort of human samples from endemic areas for both schistosome and arboviral infections, this study supports for the first time a possible association between schistosome and DENV infections. Despite the value of this proof-of-concept, the limitations of our study must be considered. Due to the unexpectedly low DENV seroprevalence, the sample size, although nearly doubled compared with our initial assumptions, was still insufficient to perform a precise risk factor analysis. Furthermore, sampling occurred in rural settings that are ideal for schistosomiasis but might be less favourable for DENV infections, which are more frequently described in urban settings [2, 34]. The use of samples from other studies limited the ad hoc sampling design, preventing the investigation of specific indicators that might have strengthened some of the results. In addition, the PRNT was conducted on a non-human cell line, so conclusions may have limited applicability to humans. Moreover, the limited availability of samples did not allow further investigations into soluble factors responsible for the observed reduction in DENV infectivity.

## Conclusions

In conclusion, our study suggests that schistosomiasis may exert a protective effect against DENV infections, as sera from infected participants significantly inhibited viral infection in vitro. By combining a well-controlled epidemiological survey with experimental laboratory-based methodologies, we shed light upon potential interactions between co-circulating infections that could influence infectious disease dynamics on the African continent. These findings open up new avenues for studies aimed at understanding the underlying mechanisms of protection and the interplay of chronic schistosomiasis and DENV.

## Supplementary Information

Supplementary material 1.

## Data Availability

Research data supporting the findings of this study are available upon reasonable request from the corresponding author.

## Abbreviations

BNITM: Bernhard Nocht Institute for Tropical Medicine
CHU: Centre Hospitalier Universitaire
*CI*: Confidence interval
COVID-19: Coronavirus disease 2019
cPOR: Crude prevalence odds ratio
CRF: Case report form
DALYs: Disability-adjusted life-years
DENV: Dengue virus
DNA: Deoxyribonucleic acid
eCRF: Electronic case report form
ELISA: Enzyme-linked immunosorbent assay
HBV: Hepatitis B virus
HCV: Hepatitis C virus
HIV: Human immunodeficiency virus
HRP: Horseradish peroxidase
IFN: Interferon
Ig: Immunoglobulin
IL: Interleukin
IQR: Interquartile range
NS: Non-structural
OD: Optical density
PCR: Polymerase chain reaction
PFU: Plaque forming units
POC-CCA: Point-of-care circulating cathodic antigen
PRNT: Plaque reduction neutralisation test
qPCR: Semi-quantitative polymerase chain reaction
Ref: Reference
RR: Risk ratio
Sneg: Schistosome-negative
Spos: Schistosome-positive
Spos-MF: Schistosome-positive with moderate liver fibrosis
Spos-NF: Schistosome-positive without liver fibrosis
Spos-SF: Schistosome-positive with severe liver fibrosis
Th: T helper cell
TNF: Tumor necrosis factor
