# Supplementary material for: An exploratory study to assess the influence of schistosomiasis on the occurrence of dengue virus in Madagascar
Source: Infect Dis Poverty. 2025 Sep 26;14:97. doi: 10.1186/s40249-025-01370-0 (PMC12465191; doi:10.1186/s40249-025-01370-0)
Supplement: Supplementary file 1 — Supplementary material 1. [file 40249_2025_1370_MOESM1_ESM.docx]

**Supplementary Table 1**: **Cytokine profiling for Atsinanana subset (comparison of three groups)**.

| **Cytokine** | **Median concentration (pg/ml) (95% *CI*)** | | ***P*-value** | **Cytokine** | **Median concentration (pg/ml) (95% *CI*)** | ***P*-value** |
| --- | --- | --- | --- | --- | --- | --- |
| **Human T-Helper Cytokine Response Panel V02** | | | | **Human Anti-Virus Response Panel** | | |
| **IL-5** |  | | 0.926 | **IFN-λ1** |  | 0.346 |
| Sneg | 30.09 (16.67–95.32) | |  | Sneg | 183.55 (181.63–213.93) |  |
| Spos with high PRNT plaque count | 41.63 (22.43–60.66) | |  | Spos with high PRNT plaque count | 181.63 (181.63–181.63) |  |
| Spos with low PRNT plaque count | 45.44 (26.24–68.14) | |  | Spos with low PRNT plaque count | 181.63 (181.63–208.99) |  |
| **IL-13** |  | | 0.296 | **IL-1β** |  | 0.369 |
| Sneg | 6.60 (6.13–15.91) | |  | Sneg | 59.50 (18.83–189.34) |  |
| Spos with high PRNT plaque count | 6.13 (5.50–8.05) | |  | Spos with high PRNT plaque count | 32.43 (22.54–47.28) |  |
| Spos with low PRNT plaque count | 6.13 (4.86–9.96) | |  | Spos with low PRNT plaque count | 32.43 (22.54–39.99) |  |
| **IL-2** |  | | 0.997 | **IFN-α2** |  | **0.044** |
| Sneg | 2.36 (1.88–4.70) | |  | Sneg | 30.70 (24.80–63.82) |  |
| Spos with high PRNT plaque count | 2.52 (1.88–3.97) | |  | Spos with high PRNT plaque count | 17.72 (8.03–22.23) |  |
| Spos with low PRNT plaque count | 2.52 (1.88–3.97) | |  | Spos with low PRNT plaque count | 19.01 (10.60–43.10) |  |
| **IL-6** |  | | 0.665 | **IL-6** |  | 0.339 |
| Sneg | 32.71 (12.84–232.97) | |  | Sneg | 15.72 (8.34–85.34) |  |
| Spos with high PRNT plaque count | 14.27 (7.08–79.41) | |  | Spos with high PRNT plaque count | 8.36 (5.08–11.38) |  |
| Spos with low PRNT plaque count | 18.62 (10.09–70.59) | |  | Spos with low PRNT plaque count | 11.37 (5.47–23. 10) |  |
| **IL-9** |  | | 0.656 | **IP-10** |  | 0.917 |
| Sneg | 8.38 (6.63–33.87) | |  | Sneg | 157.83 (127.11–190.98) |  |
| Spos with high PRNT plaque count | 8.38 (6.29–15.76) | |  | Spos with high PRNT plaque count | 154.85 (101.16–244.44) |  |
| Spos with low PRNT plaque count | 10.12 (8.38–13.90) | |  | Spos with low PRNT plaque count | 153.34 (143.14–217. 43) |  |
| **IL-10** |  | | 0.871 | **IL-10** |  | 0.108 |
| Sneg | 4.05 (2.78–6.57) | |  | Sneg | 20.16 (15.87–57.82) |  |
| Spos with high PRNT plaque count | 4.05 (3.42–6.95) | |  | Spos with high PRNT plaque count | 12.67 (7.37–18.01) |  |
| Spos with low PRNT plaque count | 5.45 (3.09–8.35) | |  | Spos with low PRNT plaque count | 19.09 (14.80–28.78) |  |
| **IFN-γ** |  | | 0.991 | **IFN-γ** |  | **0.050** |
| Sneg | 15.44 (10.01–23.52) | |  | Sneg | 164.67 (96.29–332.26) |  |
| Spos with high PRNT plaque count | 17.76 (9.99–27.10) | |  | Spos with high PRNT plaque count | 84.26 (72.23–120.83) |  |
| Spos with low PRNT plaque count | 20.01 (7.03–36.71) | |  | Spos with low PRNT plaque count | 170.97 (96.29–247.76) |  |
| **TNF-α (TNFSF2)** | |  | 0.535 | **TNF-α** |  | 0.087 |
| Sneg | 74.23 (29.02–226.97) | |  | Sneg | 119.06 (71.32–209.62) |  |
| Spos with high PRNT plaque count | 37.05 (17.97–95.35) | |  | Spos with high PRNT plaque count | 66.65 (48.40–76.00) |  |
| Spos with low PRNT plaque count | 45.07 (17.97–112.21) | |  | Spos with low PRNT plaque count | 66.65 (44.83–114.20) |  |
| **IL-17A** |  | | 0.940 | **IFN-λ2/3** |  | 0.956 |
| Sneg | 2.54 (1.92–4.69) | |  | Sneg | 210.05 (118.05–263.99) |  |
| Spos with high PRNT plaque count | 2.80 (1.54–3.33) | |  | Spos with high PRNT plaque count | 210.16 (175.02–248.77) |  |
| Spos with low PRNT plaque count | 3.06 (2.03–4.14) | |  | Spos with low PRNT plaque count | 204.68 (174.70–210.39) |  |
| **IL-17F** |  | | 0.532 | **GM-CSF** |  | 0.376 |
| Sneg | 4.88 (2.16–11.48) | |  | Sneg | 25.04 (13.63–48.01) |  |
| Spos with high PRNT plaque count | 2.35 (2.16–6.71) | |  | Spos with high PRNT plaque count | 17.06 (9.17–27.75) |  |
| Spos with low PRNT plaque count | 4.49 (2.35–10.76) | |  | Spos with low PRNT plaque count | 22.35 (13.63–53.60) |  |
| **IL-4** |  | | 0.421 | **IFN-β** |  | 0.307 |
| Sneg | 6.11 (5.20–24.61) | |  | Sneg | 130.10 (41.70–519.00) |  |
| Spos with high PRNT plaque count | 5.20 (5.20–6.38) | |  | Spos with high PRNT plaque count | 56.99 (39.79–113.88) |  |
| Spos with low PRNT plaque count | 5.20 (5.20–6.73) | |  | Spos with low PRNT plaque count | 113.88 (45.63–173.54) |  |
| **IL-22** |  | | 0.616 | **IL-8** |  | 0.763 |
| Sneg | 6.54 (3.57–14. 68) | |  | Sneg | 508.14 (31.37–4047.06) |  |
| Spos with high PRNT plaque count | 8.29 (4.85–14.34) | |  | Spos with high PRNT plaque count | 215.21 (28.08–533.54) |  |
| Spos with low PRNT plaque count | 6.80 (5.33–8.26) | |  | Spos with low PRNT plaque count | 186.11 (32.56–531. 40) |  |
|  |  | |  | **IL-12p70** |  | 0.451 |
|  |  | |  | Sneg | 8.26 (4.35–21.68) |  |
|  |  | |  | Spos with high PRNT plaque count | 5.88 (2.00–14.06) |  |
|  |  | |  | Spos with low PRNT plaque count | 11.27 (4.85–16.87) |  |

*CI* confidence interval, *Sneg* schistosome-negative, *Spos* schistosome-positive, *PRNT* plaque reduction neutralisation test.

**Supplementary Table 2**: **Cytokine profiling for Atsinanana subset (joined Schistosoma group – two group comparison)**.

| **Cytokine** | **Median concentration (pg/ml) (95% *CI*)** | | ***P*-value** | **Cytokine** | **Median concentration (pg/ml) (95% *CI*)** | ***P*-value** |
| --- | --- | --- | --- | --- | --- | --- |
| **Human T-Helper Cytokine Panel V02** | | | | **Human Anti-Virus Response Panel** | | |
| **IL-5** |  | | 0.864 | **IFN-λ1** |  | 0.165 |
| Sneg | 30.09 (16.67–116.22) | |  | Sneg | 183.55 (181.63–216.42) |  |
| Spos | 43.54 (28.17–54.98) | |  | Spos | 181.63 (181.63–181.63) |  |
| **IL-13** |  | | 0.134 | **IL-1β** |  | 0.164 |
| Sneg | 6.60 (6.13–15.91) | |  | Sneg | 59.50 (18.83–192.35) |  |
| Spos | 6.13 (5.50–6.13) | |  | Spos | 32.43 (24.53–41.85) |  |
| **IL-2** |  | | 0.974 | **IFN-α2** |  | **0.014** |
| Sneg | 2.36 (1.88–4.70) | |  | Sneg | 30.70 (22.55–63.82) |  |
| Spos | 2.52 (1.88–3.23) | |  | Spos | 18.04 (10.93–21.42) |  |
| **IL-6** |  | | 0.425 | **IL-6** |  | 0.178 |
| Sneg | 32.71 (12.84–232.97) | |  | Sneg | 15.72 (8.34–85.34) |  |
| Spos | 15.86 (8.56–48.61) | |  | Spos | 10.22 (6.88–17.36) |  |
| **IL-9** |  | | 0.551 | **IP-10** |  | 0.803 |
| Sneg | 8.38 (6.63–33.87) | |  | Sneg | 157.83 (127.11–190.98) |  |
| Spos | 8.38 (7.33–11.98) | |  | Spos | 154.09 (132.20–199.75) |  |
| **IL-10** |  | | 0.649 | **IL-10** |  | 0.107 |
| Sneg | 4.05 (2.78–6.91) | |  | Sneg | 20.16 (16.41–47.57) |  |
| Spos | 4.39 (3.42–6.14) | |  | Spos | 16.94 (11.08–21.78) |  |
| **IFN-γ** |  | | 0.913 | **IFN-γ** |  | 0.107 |
| Sneg | 15.44 (11.07–22.33) | |  | Sneg | 164.67 (96.29–332.26) |  |
| Spos | 18.89 (10.53–25.90) | |  | Spos | 105.54 (78.21–183.69) |  |
| **TNF-α (TNFSF2)** | |  | 0.270 | **TNF-α** |  | **0.029** |
| Sneg | 74.23 (29.02–201.61) | |  | Sneg | 119.06 (76.00–224.90) |  |
| Spos | 41.06 (23.50–84.90) | |  | Spos | 66.65 (48.40–85.36) |  |
| **IL-17A** |  | | 0.913 | **IFN-λ2/3** |  | 0.988 |
| Sneg | 2.54 (1.92–4.69) | |  | Sneg | 210.05 (123.11–274. 95) |  |
| Spos | 2.80 (2.16–3.46) | |  | Spos | 207.42 (180.53–215.99) |  |
| **IL-17F** |  | | 0.484 | **GM-CSF** |  | 0.661 |
| Sneg | 4.88 (2.16–11.48) | |  | Sneg | 25.04 (13.63–48.01) |  |
| Spos | 3.49 (2.25–5.15) | |  | Spos | 21.02 (12.79–27.75) |  |
| **IL-4** |  | | 0.212 | **IFN-β** |  | 0.415 |
| Sneg | 6.11 (5.20–12.92) | |  | Sneg | 130.10 (40.15–519.00) |  |
| Spos | 5.20 (5.20–5.97) | |  | Spos | 71.40 (47.67–130.34) |  |
| **IL-22** |  | | 0.522 | **IL-8** |  | 0.488 |
| Sneg | 6.54 (3.57–15.18) | |  | Sneg | 508.14 (31.37–4047.06) |  |
| Spos | 7.28 (6.30–9.15) | |  | Spos | 200.66 (38.33–437.92) |  |
|  |  | |  | **IL-12p70** |  | 0.650 |
|  | | | | Sneg | 8.26 (3.62–21.68) |  |
|  |  | |  | Spos | 9.21 (4.85–13.22) |  |

*CI* confidence interval, *Sneg* schistosome-negative, *Spos* schistosome-positive.

**Supplementary Table 3**: **Cytokine profiling for Haute Matsiatra subset (four group comparison and joined Schistosoma groups – two group comparison)**.

| **Cytokine** | **Median concentration (pg/ml) (95% *CI*)** | ***P*-value** | **Cytokine** | **Median concentration (pg/ml) (95% *CI*)** | ***P*-value** |
| --- | --- | --- | --- | --- | --- |
| **Human Anti-Virus Response Panel V02** | | | | | |
| **Four groups** | | | **Two groups** | | |
| **IFN-λ1** |  | 0.869 | **IFN-λ1** |  | 0.426 |
| Sneg | 99.41 (56.07–252.23) |  | Sneg | 99.41 (56.07–252.23) |  |
| Spos-NF | 105.68 (66.81–311.75) |  | Spos | 124.26 (79.97–225.29) |  |
| Spos-MF | 142.13 (67.46–272.45) |  |  |  |  |
| Spos-SF | 118.23 (79.97–262.40) |  |  |  |  |
| **IL-1β** |  | 0.954 | **IL-1β** |  | 0.635 |
| Sneg | 16.33 (8.00–124.75) |  | Sneg | 16.33 (8.00–124.75) |  |
| Spos-NF | 18.91 (9.92–115.31) |  | Spos | 20.23 (14.97–35.34) |  |
| Spos-MF | 21.58 (12.46–34.32) |  |  |  |  |
| Spos-SF | 20.23 (11.17–189.89) |  |  |  |  |
| **IFN-α2** |  | 0.589 | **IFN-α2** |  | 0.217 |
| Sneg | 15.03 (5.03–44.60) |  | Sneg | 15.03 (5.03–38.90) |  |
| Spos-NF | 23.30 (11.90–65.80) |  | Spos | 23.3 (16.68–32.78) |  |
| Spos-MF | 25.50 (18.53–42.80) |  |  |  |  |
| Spos-SF | 21.85 (7.16–39.98) |  |  |  |  |
| **IL-6** |  | 0.560 | **IL-6** |  | 0.658 |
| Sneg | 102.00 (72.96–221.95) |  | Sneg | 102.00 (72.96–221.95) |  |
| Spos-NF | 109.42 (75.94–205.64) |  | Spos | 125.09 (94.17–164.92) |  |
| Spos-MF | 164.92 (94.17–314.58) |  |  |  |  |
| Spos-SF | 109.83 (72.96–189.09) |  |  |  |  |
| **IP-10** |  | 0.928 | **IP-10** |  | 0.623 |
| Sneg | 110.53 (88.35–131.37) |  | Sneg | 110.53 (88.35–131.37) |  |
| Spos-NF | 125.55 (67.47–166. 72) |  | Spos | 119.73 (101.32–142.55) |  |
| Spos-MF | 119.62 (81.24–145.28) |  |  |  |  |
| Spos-SF | 119.73 (74.58–169.36) |  |  |  |  |
| **IL-10** |  | 0.202 | **IL-10** |  | 0.165 |
| Sneg | 15.54 (10.76–39.79) |  | Sneg | 15.54 (10.76–39.01) |  |
| Spos-NF | 43.05 (25.17–69.16) |  | Spos | 25.98 (24.36–38.16) |  |
| Spos-MF | 25.17 (21.94–44.28) |  |  |  |  |
| Spos-SF | 25.17 (10.76–36.53) |  |  |  |  |
| **IFN-γ** |  | 0.096 | **IFN-γ** |  | 0.150 |
| Sneg | 76.44 (68.00–184.44) |  | Sneg | 76.44 (68.00–184.44) |  |
| Spos-NF | 125.24 (90.56–280.95) |  | Spos | 119.40 (90.56–196.36) |  |
| Spos-MF | 190.40 (102.06–238.51) |  |  |  |  |
| Spos-SF | 79.28 (73.61–148.72) |  |  |  |  |
| **TNF-α** |  | 0.242 | **TNF-α** |  | 0.101 |
| Sneg | 178.13 (126.32–304.34) |  | Sneg | 178.13 (126.32–304.34) |  |
| Spos-NF | 146.89 (107.01–194.46) |  | Spos | 130.13 (114.82–146.89) |  |
| Spos-MF | 133.86 (104.97–157.34) |  |  |  |  |
| Spos-SF | 126.32 (84.58–215.69) |  |  |  |  |
| **IFN-λ2** |  | 0.259 | **IFN-λ2** |  | 1 |
| Sneg | 342.03 (249.65–412.90) |  | Sneg | 342.02 (274.90–412.90) |  |
| Spos-NF | 330.00 (260.30–403.65) |  | Spos | 344.55 (305.80–368.15) |  |
| Spos-MF | 339.93 (205.60–358.15) |  |  |  |  |
| Spos-SF | 368.15 (291.05–499.00) |  |  |  |  |
| **GM-CSF** |  | 0.525 | **GM-CSF** |  | 0.773 |
| Sneg | 21.83 (11.00–64.86) |  | Sneg | 21.83 (11.00–49.21) |  |
| Spos-NF | 27.01 (20.57–58.11) |  | Spos | 27.01 (16.38–36.87) |  |
| Spos-MF | 16.38 (12.02–32.46) |  |  |  |  |
| Spos-SF | 40.18 (14.34–44.66) |  |  |  |  |
| **IFN-β** |  | 0.570 | **IFN-β** |  | 0.973 |
| Sneg | 72.46 (35.28–129.30) |  | Sneg | 72.46 (35.28–113.14) |  |
| Spos-NF | 109.03 (35.28–153.88) |  | Spos | 64.17 (38.71–133.43) |  |
| Spos-MF | 104.81 (35.28–181.88) |  |  |  |  |
| Spos-SF | 58.64 (33.62–112.98) |  |  |  |  |
| **IL-8** |  | 0.430 | **IL-8** |  | 0.607 |
| Sneg | 624.68 (49.63–3072.71) |  | Sneg | 624.68 (49.63–3072.71) |  |
| Spos-NF | 229.11 (63.39–7516.95) |  | Spos | 306.36 (57.27–731.06) |  |
| Spos-MF | 167.65 (34.48–479.40) |  |  |  |  |
| Spos-SF | 648.12 (43.55–6314.96) |  |  |  |  |
| **IL-12p70** |  | 0.639 | **IL-12p70** |  | 0.294 |
| Sneg | 5.95 (1.74–18.10) |  | Sneg | 5.95 (1.59–19.56) |  |
| Spos-NF | 14.29 (2.43–25.17) |  | Spos | 10.71 (5.34–15.28) |  |
| Spos-MF | 9.09 (5.03–12.49) |  |  |  |  |
| Spos-SF | 10.38 (1.31–32.07) |  |  |  |  |

*CI* confidence interval, *Sneg* schistosome-negative, *Spos* schistosome-positive, *Spos-NF* schistosome-positive without liver fibrosis, *Spos-MF* schistosome-positive with moderate liver fibrosis, *Spos-SF* schistosome-positive with severe liver fibrosis.


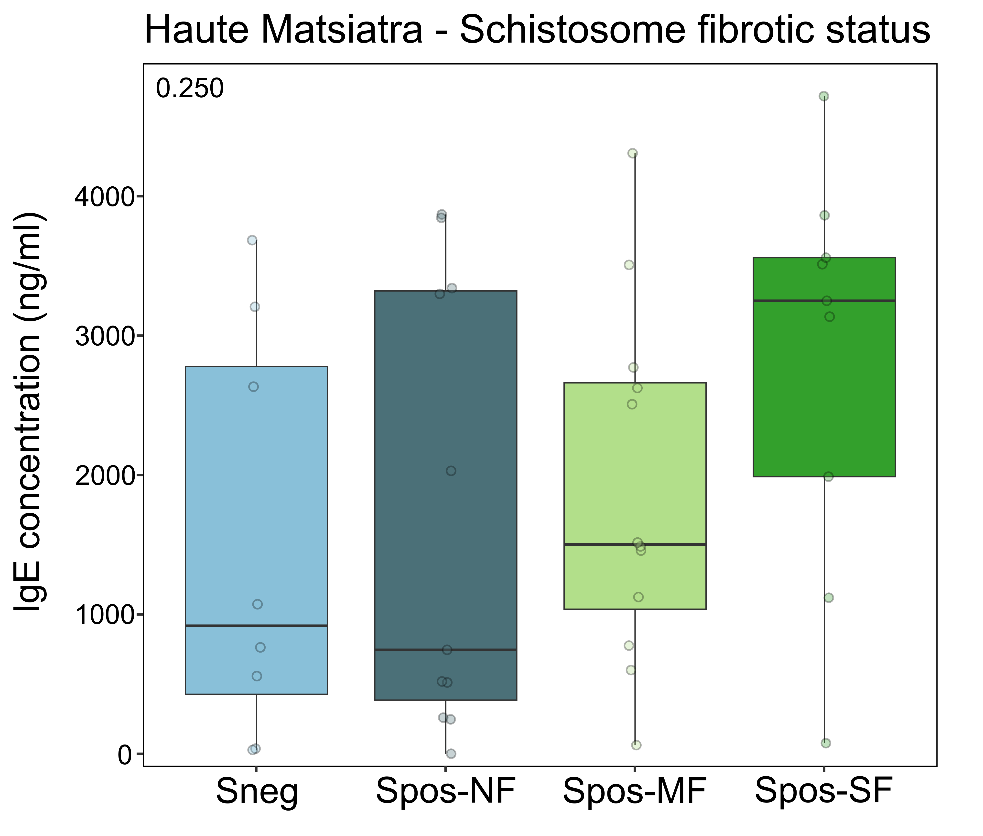


**Supplementary Figure 1**: **IgE concentrations among a subset of plasma from Haute Matsiatra**. A subset of 40 samples (*n* Sneg = 8, *n* Spos-NF = 11, *n* Spos-MF = 12, *n* Spos-SF = 9) was subjected to the Human IgE ELISA (Invitrogen, Waltham, USA). *P*-value was assessed using Kruskal-Wallis test. *Sneg* schistosome-negative, *Spos-NF* schistosome-positive without liver fibrosis, *Spos-MF* schistosome-positive with moderate liver fibrosis, *Spos-SF* schistosome-positive with severe fibrosis, *ELISA* enzyme-linked immunosorbent assay.
